# Multi-domain Features of the Non-phase-locked Component of Interest Extracted from ERP Data by Tensor Decomposition

**DOI:** 10.1007/s10548-019-00750-8

**Published:** 2019-12-26

**Authors:** Guanghui Zhang, Chi Zhang, Shuo Cao, Xue Xia, Xin Tan, Lichengxi Si, Chenxin Wang, Xiaochun Wang, Chenglin Zhou, Tapani Ristaniemi, Fengyu Cong

**Affiliations:** 1 School of Biomedical Engineering, Faculty of Electronic Information and Electrical Engineering, Dalian University of Technology, Dalian, 116024 China; 2 Faculty of Information Technology, University of Jyväskylä, 40100 Jyvaskyla, Finland; 3 School of Foreign Languages, Dalian University of Technology, Dalian, 116024 China; 4 School of Psychology, Shanghai University of Sport, Shanghai, 200438 China

**Keywords:** ERP, Mother wavelet, Tensor decomposition, Time–frequency analysis, Non-phase locked

## Abstract

The waveform in the time domain, spectrum in the frequency domain, and topography in the space domain of component(s) of interest are the fundamental indices in neuroscience research. Despite the application of time–frequency analysis (TFA) to extract the temporal and spectral characteristics of non-phase-locked component (NPLC) of interest simultaneously, the statistical results are not always expectedly satisfying, in that the spatial information is not considered. Complex Morlet wavelet transform is widely applied to TFA of event-related-potential (ERP) data, and mother wavelet (which should be firstly defined by center frequency and bandwidth (CFBW) before using the method to TFA of ERP data) influences the time–frequency results. In this study, an optimal set of CFBW was firstly selected from the number sets of CFBW, to further analyze for TFA of the ERP data in a cognitive experiment paradigm of emotion (Anger and Neutral) and task (Go and Nogo). Then tensor decomposition algorithm was introduced to investigate the NPLC of interest from the fourth-order tensor. Compared with the TFA results which only revealed a significant difference between Go and Nogo task condition, the tensor-based analysis showed significant interaction effect between emotion and task. Moreover, significant differences were found in both emotion and task conditions through tensor decomposition. In addition, the statistical results of TFA would be affected by the selected region of interest (ROI), whereas those of the proposed method were not subject to ROI. Hence, this study demonstrated that tensor decomposition method was effective in extracting NPLC, by considering spatial information simultaneously as the potential to explore the brain mechanisms related to experimental design.

## Introduction

Electroencephalogram (EEG) has been extensively used in neuroscience since Berger Hans first recorded it from the human cerebral cortex in 1929 (Berger 1929). In early studies, most researchers mainly focused on the amplitude of an individual waveform in the time domain. With the introduction of computers, besides waveform, spectral and spatial characteristics of the component(s) of interest (COI) for group-averaged EEG/event-related-potential (ERP) data are analyzed (Luck 2014). They found that ERP components could be evoked from the related experiments, and have specific temporal, spectral and spatial characteristics. For instance, when words and other meaningful (or potentially meaningful) excitations include visual and auditory words, sign language signs, pictures, faces, environmental sounds, and smells are used for experimental stimuli, N400, a negative waveform which reaches a peak around 400 ms after stimulus onset and can extend the time window from 250 to 500 ms, can be discovered (Kutas and Federmeier 2000, 2011; Kutas and Hillyard 1980). Meanwhile, it is typically maximal over centro-parietal electrodesites. Therefore, all the temporal, spectral and spatial properties of ERP component(s) are useful for the investigation of brain mechanisms in cognitive processes and these properties may be coupled. Several techniques have been developed for ERP data processing and analyze to dig out the potential information in the cognitive processes, such as time domain analysis and time–frequency analysis (TFA).

Most previous studies focus on time domain analysis. The conventional method averages several single-trial data of the same stimulus in the time domain to obtain ERP components. The advantage of this method is that the energy of ERP is enhanced, with the amplitude of spontaneous EEG and noise extremely reduced (Cohen 2014). Some advanced signal processing and analyze methods also have been developed to extract COI from group-averaged ERP data such as Independent Component Analysis (ICA) (Hyvärinen 2013; Jung et al. 2000) and Principal Component Analysis (PCA) (Dien 2010a, b, 2012; Dien et al. 2005; Möcks and Verleger 1985; Kawaguchi et al. 2013). However, the main drawback of the time domain analysis is that it cannot reveal COI changes in the frequency domain over time so that the pivotal rhythm (or oscillation) information is neglected.

To extract the temporal and spectral characteristics of ERP component(s) simultaneously, some researchers use short-time Fourier transform (STFT) or wavelet transform algorithm (WTA) to convert time domain signals into time–frequency domain signals. There are two strategies for TFA of ERP data. One is evoked method in which multi-trial data are averaged before the computation of the time–frequency transforms of averaged ERP data. The event-related oscillations (EROs) obtained by this type of TFA are extremely phase-locked to stimulus onset because of the simultaneous co-occurrence of enhanced EROs. The time locked and phase locked component (TLPLC) can be obtained and it is called evoked brain activity. The other one is based on averaging the time–frequency transforms of every single-trial. Both TLPLC and non-phase-locked component (NPLC) are summed up so that it refers to all-brain activities. And this strategy is considered as the induced method (Herrmann et al. 2004, 2014; Tallon-Baudry and Bertrand 1999). The induced method has two superiorities over time domain analysis or evoked method. The first one is that it can simultaneously exploit the temporal and spectral properties of an ERP component and reveal additional NPLC activity. For the other one, the results are non-negative, which means that it can avoid the amplitude of COI being cancelled out in the averaged ERP data, if they are randomly distributed for each single-trial in the time domain (Cong et al. 2015b). The TLPLC can be obtained by time domain analysis and evoked method, whereas NPLC is generated by averaging the time–frequency transforms of every trial and this type ERO is evoked by some high-order processes (David et al. 2006; Singer and Gray 1995). Meanwhile, as described in the study (David et al. 2006), TLPLC reflects some stimulus locked event related response, while NPLC might be evoked by nonlinear and autonomous mechanisms. In short, the neuronal processes and mechanisms of TLPLC and NPLC are different (David et al. 2006).

Since Tallon Baudry et al. proposed the NPLC-oriented TF method in 1996 (Tallon-Baudry et al. 1996), it has been widely used in the fields of cognitive neuroscience and medicine, such as Parkinson’s disease (Wiesman et al. 2016), depression (Shaw et al. 2013), children sleep (Piantoni et al. 2013), and language cognition (Araki et al. 2016; Kielar et al. 2015; Wang et al. 2012). Hence, NPLC includes significant information of all-brain activities. However, the spatial information is still not utilized in TFA and sometimes statistically significant results cannot be obtained by TFA, which poses some challenges for the exploration of brain mechanisms. In such a context, we propose a NPLC-oriented tensor decomposition analysis of ERP data. Tensor decomposition exploits the interaction among modes. Firstly defined in the mathematics field (Hitchcock 1927), it has been extensively applied in the fields of psychometrics and chemometrics for multi-mode data analysis (Kroonenberg 2008; Smilde et al. 2004).

Aiming to overcome the shortcomings of time domain analysis and TFA, some researchers have attempted to use Canonical Polyadic decomposition (CPD) (Hitchcock 1927) to extract multi-domain features of COI simultaneously from high-order tensor composed of time–frequency results. Here, the high-order tensor is a fourth-order tensor. The order of the fourth-order tensor represents the number of its “ways”, “dimensions”, “domains”, or “modes”, which includes four modes: frequency, time, channels/space, and subjects-stimuli/conditions (Zhou et al. 2016). The component is selected if its temporal, spectral, and spatial components are consistent with characteristics of COI in the time, frequency, and space domains, and then its multi-domain feature mode (the last mode) is applied to statistical analysis (Cong et al. 2012c, 2013, 2014). Despite the use of tensor decomposition algorithm to extract TLPLC of interest (Cong et al. 2012c), NPLC from all brain activities has not been investigated with tensor-based multi-mode analysis (more than three modes).

**Fig. 1:**
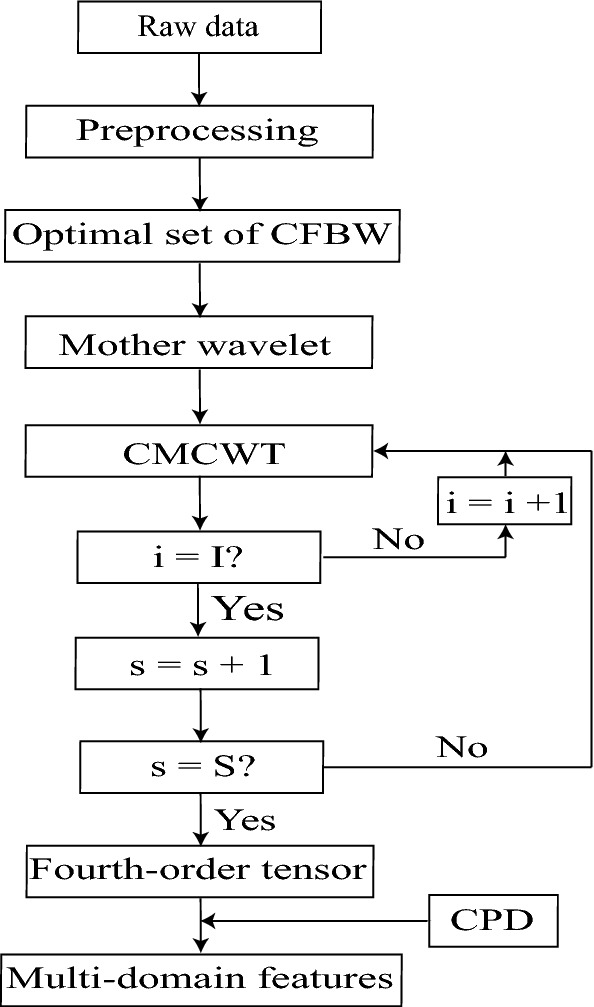
The flow of tensor-based method for ERP data analysis. *S* is the number of subjects-stimuli/conditions; *I* represents the number of channels

**Fig. 2:**
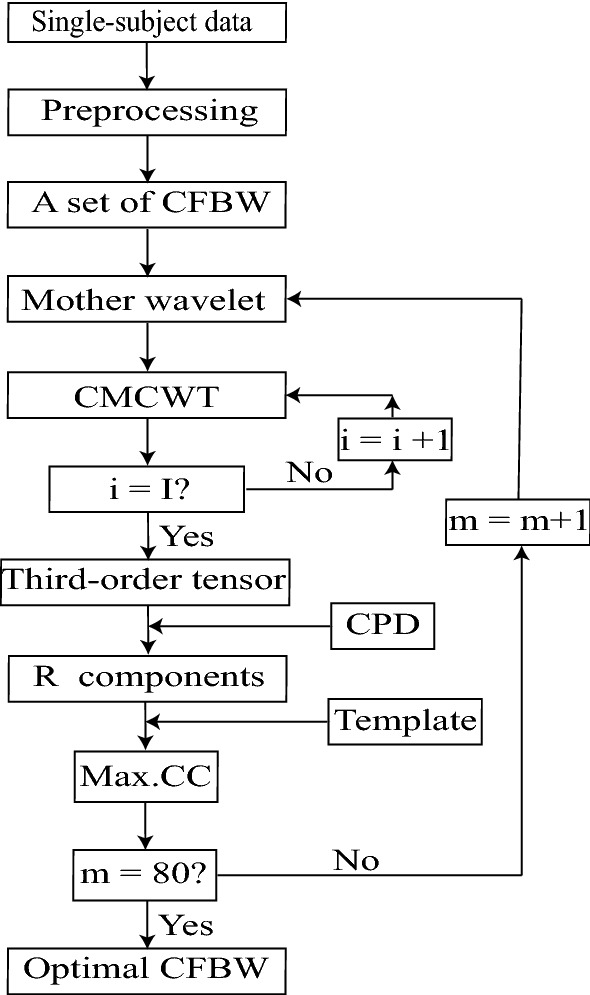
Optimal CFBW set selection. *I* and 80 are the number of channels and total number of sets of CFBW, respectively. *Max.CC* represents maximum correlation coefficient

There are two problems to be solved before extracting NPLC. For one thing, when referring to TFA of ERP data, it is typically calculated by WTA (Herrmann et al. 2014; Tallon-Baudry and Bertrand 1999; Tallon-Baudry et al. 1996, 1997, 1998). Some researchers also use STFT for TFA of ERP data, but the central idea is similar to WTA (Hu et al. 2014). When WTA is used for TFA of ERP data, a mother wavelet should be firstly defined by a set of center frequency and bandwidth (CFBW). Since the differences of CFBW may result in divergent time–frequency results, different CFBW should be attempted for an optimal time–frequency result (Zhang et al. 2017). For another, NPLC is mixed together with other components (Jung et al. 2000). The key problem is how to separate NPLC from mixed signals. This study is dedicated to the investigation of these issues and the following steps are used for implementing the idea of NPLC-oriented tensor decomposition analysis. After ERP data preprocessing, CFBW are optimized by selecting from 80 sets of CFBW to define a mother wavelet for complex morlet continuous wavelet transform (CMCWT), which is used to solve the first problem as mentioned above (as shown in Fig. 2). The induced method was conducted to convert the time domain signals of every participant into the time–frequency domain signals, so that the fourth-order tensor was formed. Subsequently, the temporal components, spectral components, spatial components, and features of subjects-stimuli/conditions mode of NPLC are extracted simultaneously by CPD from the fourth-order tensor (to solve the second problem as mentioned above). Finally, a comparison was made of the diversity between NPLC extracted by CPD and TFA in the temporal component, spectral component, spatial component, and repeated measure analysis of variance (rm-ANOVA) results (the flow of data processing and analyze as shown in Fig. 1).

## Methods

### Participants and EEG Data Acquisition

Fifteen college students were recruited to participate as paid volunteers from Shanghai University of Sports in China. Seven were females and eight were males (Mean age: 20.8; Std: 1.4). All the participants were right-handed, presented with normal or corrected to normal visual acuity and they did not know or see the experimental paradigm before the experiment. Previous studies reported that anger appeared to be an important factor in human behavior (Denny and Siemer 2012) and the emotion Go/Nogo task was used in a great number of studies to explore the underlying mechanisms (Goldstein et al. 2007; Shafritz et al. 2006; Verona et al. 2012; Yu et al. 2014). Following this line, in this study, all participants were required to participate in emotion (Anger and Neutral) Go/Nogo task. The details of the experiment materials and the paradigm can be found in our previous research (Xia et al. 2018). EEG recordings at 64 locations were collected according to the standard 10–20 system. The EEG data were referenced online against the FCz electrode and grounded at the AFz electrode. Meanwhile, a vertical electrooculogram was obtained below the left eye, and the horizontal electrooculogram was obtained at the outer canthus of the right eye. Impedances were less than 5 $\hbox {k}\Omega$. The BrainAmp amplifier and BrainVision Recorder 2.0 system (Brain Products GmbH, Germany) were used to record electrical activity for each participant with a 500 Hz sampling rate and the data were filtered between 0.01 and 100 Hz by a BrainAmp amplifier.

### Data Preprocessing

The preprocessing of EEG was conducted in two sorts of software. Firstly, by the Analyzer 2.0 system (Brain Products), the FCz electrode was restored when the data were re-referenced offline to an average of both posterior ear papillae (TP9 and TP10) for each participant (Debener et al. 2012). Subsequently, using the EEGLAB toolbox (Delorme and Makeig 2004) running in the MATLAB environment (MathWorks, Natick, MA), the data preprocessing was performed offline. The EEG signals were filtered offline with a 45–55 Hz notch infinite impulse response (IIR) (Delorme and Makeig 2004; Guan et al. 2004; Kropotov 2010; Lopez-Calderon and Luck 2014; Nishida et al. 1993; Widmann et al. 2015) filter (to remove line noise), a high-pass IIR filter of 0.2 Hz and a low-pass IIR filter with 30 Hz, respectively. Furthermore, the filtered continuous recordings were epoched from 200 ms before the stimulus onset to 1000 ms after the stimulus onset. Epochs/trials whose maximum magnitude exceeded $100\,{\upmu V}$ were excluded ($5.7\%$ of epochs/trials were rejected) and then remaining epochs were baseline corrected. Considering the COI is below 30 Hz and in order to reduce the impact of low-frequency components, a band-pass filter with 3–30 Hz based on Fast Fourier Transformation (FFT) was applied to filter the single-trial data (Cong et al. 2015b). Taking into account the diversity of bad channels of each participant, 22 bad channels were removed for all participants. They were identified based on the data distribution and variance of channels, by using the EEGLAB’s function-pop_rejchan (Delorme and Makeig 2004) and the FASTER toolbox (Nolan et al. 2010).

### Time–Frequency Analysis

#### Complex Morlet Continuous Wavelet Transform

STFT and WTA are two common algorithms in the TFA of ERP data. STFT has been extensively utilized for TFA of ERP data since it is proposed by Potter in 1947 (Araki et al. 2016; Cohen 1989; Ehm et al. 2011; Fumuro et al. 2015; Kauppi et al. 2013). This method is to calculate the Fourier transform of the windowed signals, which are approximately stationary over the window. However, the length of the window is the same for all frequencies. If the length of the window is too long, it will lead to low time-resolution at higher frequencies and low frequency-resolution at lower frequencies. In contrast, when the window length is relatively shorter, it will present the opposite results. Compared with STFT, the wavelet transform uses short windows at high frequencies and long windows at low frequencies (Rioul and Vetterli 1991; Peng and Chu 2004). That is to say, the wavelet transform is more adapted to TFA of non-stationary signals, for example, EEG/ERP data (Peng and Chu 2004). Therefore, WTA is used to achieve a trade-off between time-resolution and frequency-resolution in this study.

When the length of discrete sequence signal $\varvec{y}(t)$ is $T(t=0,1,2,{\ldots }, T-1)$, then the wavelet transform can be expressed as (Zhang et al. 2017):

1 $$\begin{aligned} \varvec{Y}(a,t_0)=\frac{1}{\sqrt{ \mid a \mid }} \sum _{t=0}^{T-1}\varvec{y}(t) \varPhi \left( \frac{t-t_0}{ a }\right) . \end{aligned}$$

In the above formula, $\varPhi (\frac{t-t_0}{ a })$ is the mother wavelet. *a* and $t_0$ are called scaling and shifting parameters, respectively. In this study, the complex Morlet Wavelet is defined as the mother wavelet (Tallon-Baudry and Bertrand 1999; Tallon-Baudry et al. 1996, 1997, 1998; Bertrand and Tallon-Baudry 2000; Simões et al. 2003; Lachaux et al. 2005; Li et al. 2019; Xia et al. 2019):

2 $$\begin{aligned} \varPhi (t,f_c)=\frac{1}{\sqrt{ \pi {f_b}^2 }}e^{i2 \pi f_ct} e^{\frac{-t^2}{2{\ f_b}^2}}, \end{aligned}$$

where $f_b$ and $f_c$ stand for bandwidth and center frequency, respectively. And a gaussian shape respectively in the time domain and frequency domain around its $f_c$ can be obtained (Zhang et al. 2017).

A wavelet family is characterized by a constant ratio (Tallon-Baudry et al. 1996):

3 $$\begin{aligned} K = \frac{f_c}{{f_b}_f} = 2{\pi }{f_b}{f_c}. \end{aligned}$$

In this formula,${\ {f_b}_f}= \frac{1}{2{\pi }{f_b}}$, *K* should be more than 5 (Zhang et al. 2017).

Given an ERP data $\varvec{x}_{c,n}(t)$, *c* and *n* are the number of the electrodes/sensors and trials, respectively. The definition of induced method can be given (Herrmann et al. 2005) by the following equation:

4 $$\begin{aligned} \varvec{ERSP_{av}}(t,f) = \frac{1}{N}\sum _{n=1}^{N} {{\mid {\varvec{X}_{c,n}(t,f)} \mid }^2}. \end{aligned}$$

In Eq. 4, ${{\mid {\varvec{X}_{c,n}(t,f)} \mid }^2}$ represent the power values of ERP data in *cth* electrode and *nth* trial.

#### Selection of an Optimal Set of CFBW for CMCWT

As shown in our previous study (Zhang et al. 2017), different parametric settings may result in divergent time–frequency results. CMCWT [the MATLAB function (Daubechies 1992; Mallat 1999)] was used under the MATLAB environment for the TFA of ERP data with an optimal set of CFBW selected from a number of sets of CFBW (as shown in Fig. 2). The specific steps are as below.

Eighty sets were generated through different combinations of center frequency $(f_c)$ and bandwidth $(f_b)$ under the constraint of $K > 5$. *K* is the constant ratio in Eq. 3. The combinations are as follows: when ${f_b}_1 = 0.1, f_c = 9, 10$, respectively; when ${f_b}_2 = 0.2, f_c =5, 6, 7, 8, 9, 10$, respectively; when ${f_b}_3 = 0.3, f_c = 3, 4, 5, 6, 7,$ 8, 9, 10, respectively; when ${f_b}_4 = 0.4, f_c = 3, 4, 5, 6, 7,$ 8, 9, 10, respectively; when ${f_b}_5 = 0.5, f_c =2, 3, 4, 5, 6, 7,$ 8, 9, 10, respectively; when ${f_b}_6 = 0.6, f_c =2, 3, 4, 5, 6,$ 7, 8, 9, 10, respectively; when ${f_b}_7 = 0.7, f_c =2, 3, 4, 5, 6, 7, 8,$ 9, 10, respectively; when ${f_b}_8 = 0.8, f_c = 2, 3, 4, 5, 6, 7, 8, 9, 10$, respectively; when ${f_b}_9 = 0.9, f_c =1, 2,3, 4, 5,$ 6, 7, 8, 9, 10, respectively; when ${f_b}_{10} = 1, f_c =1, 2, 3, 4, 5,$ 6, 7, 8, 9, 10, respectively.

The CFBW in each set was applied to the TFA (see “Complex Morlet Continuous Wavelet Transform” section) of the same single subject data under one condition.

Each set of CFBW corresponded to a time–frequency representation (TFR) and topography (obtained by averaging the same region of interest, the time window from 300–600 ms and the frequency range from 3 to 7 Hz for all sets of CFBW) obtained by TFA. Meanwhile, third-order tensor including frequency, time, and channels can be composed by the time–frequency results of all sets of CFBW, respectively.

A typical topographical distribution of time–frequency results was referred to as the template. For instance, when ${f_b} = 1$, the value of ${f_c}$ can be respectively set as 1, 2, 3, 4, 5, 6, 7, 8, 9,  and 10. The topographical distribution of $f_{c_4}= 4$ was finally chosen as the template $T_t{_e}{_m}{_p}{_l}{_a}{_t}{_e} ({f_b}_{10}, f_{c_4})$, based on the comparison of its topography and TFR with those of other sets of CFBW. That is to say, the time-resolution and frequency-resolution of its TFR are better than other sets of CFBW, and the template could represent most of the topographic maps of all sets of CFBW. After defining $f_{c_n}$, the correlation coefficients (CCs) between the template ($T_t{_e}{_m}{_p}{_l}{_a}{_t}{_e}$) and spatial components $s_r({f_b}_{10},f_{c_n})$ obtained by CPD were calculated by the following equations (*R* components were extracted in each mode based on the method as described in our previous studies (Cong et al. 2012a, 2015a), the detail of extracted number of every set of CFBW is shown in Table 1).

5 $$\begin{aligned} Y({f_b}_{10},f_{c_n},r)=\rho (s_r({f_b}_{10},f_{c_n}), T_t{_e}{_m}{_p}{_l}{_a}{_t}{_e}({f_b}_{10},f_{c_4})), \end{aligned}$$

where $r = 1, 2, {\ldots } , R; n = 1, 2, {\ldots }, 10$; *Y* is the *CC* of each component for every set of CFBW.

6 $$\begin{aligned}&\rho (s_r,T_t{_e}{_m}{_p}{_l}{_a}{_t}{_e})\nonumber \\&\quad =\frac{{\sum _{i=1}^{I}}{({s^i_r-{\overline{s_r}}})}{({T^{i}_{template}} -{\overline{T_{template}}})}}{{\sqrt{{\left( {\sum _{i=1}^{I}} {s^i_r-{\overline{s_r}}}\right) ^2}}}{\sqrt{{\sum _{i=1}^{I}} ({T^{i}_{template}}-{\overline{T_{template}}}})^2}}, \end{aligned}$$

where $\rho \in (-1,1)$ represents the CC between $s_r$ and $T_{template}$; *I* is the number of channels; $T^{i}_{template}$ and $s^i_{r}$ are the value of each channel for $T_{template}$ and $s_{r}$, respectively; $\overline{T_{template}}$ and $\overline{s_{r}}$ are the mean value of $T_{template}$ and $s_{r}$, respectively.

Subsequently, the maximal *CC* for each set of parameters was chosen as:

7 $$\begin{aligned} q({f_b}_{10},f_{c_n},r)= max(Y({f_b}_{10},f_{c_n},1), Y({f_b}_{10},f_{c_n},2),\ldots ,Y({f_b}_{10},f_{c_n},R)). \end{aligned}$$

Then, the corresponding *rth* components with the maximum *CC* were obtained. The same procedures were applied to the other sets of CFBW.

**Fig. 3:**
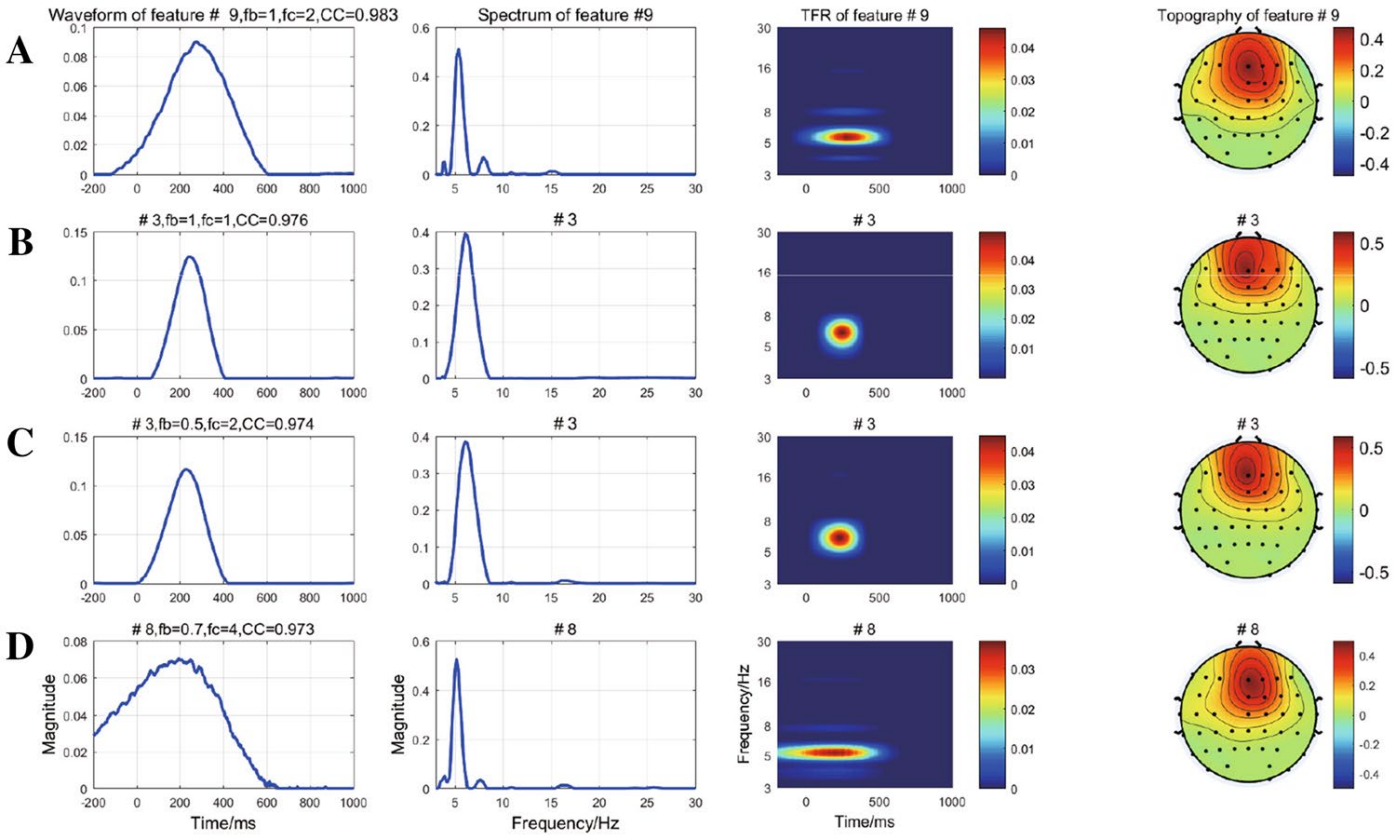
**a**–**d** The multi-domain components with the first four maximum CCs of four sets of CFBW (${f_b} = 1$, ${f_c}=2$; ${f_b} = 1$, ${f_c}=1$; ${f_b} =0.5$, ${f_c}=2$; ${f_b} = 0.7$, ${f_c}=4$), respectively

Finally, an optimal set of CFBW was selected. Take the mutil-domain components of the first four maximum CCs of four sets of CFBW as example, as shown in Fig. 3. Comparing the corresponding waveforms of the maximum *CCs* of the first four sets of CFBW, the set of ${f_b} = 0.7, {f_c} = 4$ was firstly discarded, because the COI was evoked after the stimulus onset. Then, we considered that the period of waveform of COI was relatively narrow in the time domain (usually not more than one second) and there were few irrelevant spikes for waveform and spectrum of multi-domain features extracted by CPD. ${f_b} = 1$ and ${f_c} = 1$ were used to define the mother wavelet.

**Table 1:** The extracted number in each mode of every set of CFBW

$f_c$	$f_b$									
	0.1	0.2	0.3	0.4	0.5	0.6	0.7	0.8	0.9	1
1	–	–	–	–	–	–	–	–	36	28
2	–	–	–	–	35	45	25	42	35	32
3	–	–	51	43	32	34	28	25	45	45
4	–	–	28	50	45	42	30	36	46	42
5	–	25	44	46	25	35	25	25	40	25
6	–	37	52	35	20	45	40	30	30	32
7	–	34	37	30	42	40	28	42	42	45
8	–	26	26	36	31	36	36	30	45	28
9	25	40	44	25	40	25	35	40	33	30
10	45	36	38	24	42	35	35	48	30	45

### Tensor Decomposition Algorithm

In an ERP experiment, there should be at least three modes including time, channels/space, and subjects-stimuli/conditions. When the time-domain data are converted into the time–frequency domain, a fourth-order tensor including time, frequency, channels/space, and subjects-stimuli/conditions can be formed. Moreover, EEG data are used to identify common activities over subjects. It is necessary and interesting to study the interaction among modes, such as time, frequency, and channels/space modes. Here, CPD (Hitchcock 1927; Cong et al. 2015a) is applied to extract COI from the high-order tensor.

Given an *N*th-order tensor $\underline{\varvec{X}} \in {\mathcal {{R}}}^{I_1 \times I_2 \times \cdots \times I_N }$, the CPD can be defined as:

8 $$\begin{aligned} \underline{\varvec{X}} \approx \sum _{r=1}^{R}\varvec{u}^{(1)}_r \circ \varvec{u}^{(2)}_r \circ \cdots \circ \varvec{u}^{(N)}_r + \varvec{E} =\underline{\widehat{\varvec{X}}}+\varvec{E} \approx \underline{\widehat{\varvec{X}}}. \end{aligned}$$

In Eq. 8, $$\underline{\widehat{\varvec{X}}}$$ approximates the high-order tensor $$\underline{\varvec{X}}$$; $$\varvec{E} \in {\mathcal {R}}^{I_1 \times I_2 \times \cdots \times I_N }$$ is a *Nth*-order error tensor, whose sizes of all dimensions are the same as $$\underline{\varvec{X}}$$; $$\Vert \varvec{u}^{(n)}_r \Vert _{2} = 1$$$$(n = 1,2,{\ldots }, N-1)$$. $$\varvec{U}^{(n)} = [ \varvec{u}^{(1)}_r \circ \varvec{u}^{(2)}_r \circ \cdots \circ \varvec{u}^{(N)}_r ]$$ represents a component matrix for mode $$\# n$$, and $$n = 1,2,{\ldots }, N.$$

In this study, the fourth-order tensor consisted of the time–frequency results. It can be extracted by CPD (Cong et al. 2014, 2012d):

9 $$\begin{aligned} \underline{\varvec{X}} \approx \sum _{r=1}^{R}\varvec{u}^{(f)}_r \circ \varvec{u}^{(t)}_r \circ \varvec{u}^{(s)}_r \circ \varvec{f}_r =\underline{\varvec{I}} \times _{1} {\varvec{U}^{(f)}} \times _{2} {\varvec{U}^{(t)}} \times _{3}{\varvec{U}^{(s)}}\times _{4} \varvec{F}. \end{aligned}$$

In the above formula, $\underline{\varvec{I}}$ is a diagonal tensor, the value of every element of its super-diagonal is equal to one; the component matrix $\varvec{F}$ contains the multi-domain features of *R* brain activity (*R* components are extracted in each mode), and each column corresponds to one feature. The component matrix corresponds to the *rth* components in the time domain ($\varvec{u}^{(t)}_r$), frequency domain ($\varvec{u}^{(f)}_r$) and space domain ($\varvec{u}^{(s)}_r$), respectively. Those components reveal the properties of the *rth* multi-domain feature in the domains as well (Cong et al. 2015a, 2012a). In the CPD, the *r*th temporal component, *r*th spectral component, and *r*th spatial component are interrelated, but neither of them is associated with other multi-domain components (Cong et al. 2015a). Meanwhile, combining with the generative mechanisms of NPLC of interest, CPD is selected to extract NPLC of interest from the fouth-order tensor. According to Eqs. 1 and 4, the time–frequency transforms are obtained by calculating the product of the constant and the sum of the square of the absolute value of the convolution between signals and the mother wavelet. Therefore, the elements of the high-order tensor are nonnegative in the study. In our previous study (Cong et al. 2014), the fourth-order tensor (the size of last mode is conditions by groups by subjects) is formed to find the discrepancy of cognitive processes between the two groups under every condition. Likewise, our interest is to identify the differences between emotion factor under Go/Nogo tasks by calculating statistical results of the features of last mode extracted from the fourth-order tensor (frequency by time by channels/space by subjects-stimuli/conditions: $30 \times 600 \times 42 \times (15 \times 4)$).

## Results

### Time–Frequency Analysis

**Fig. 4:**
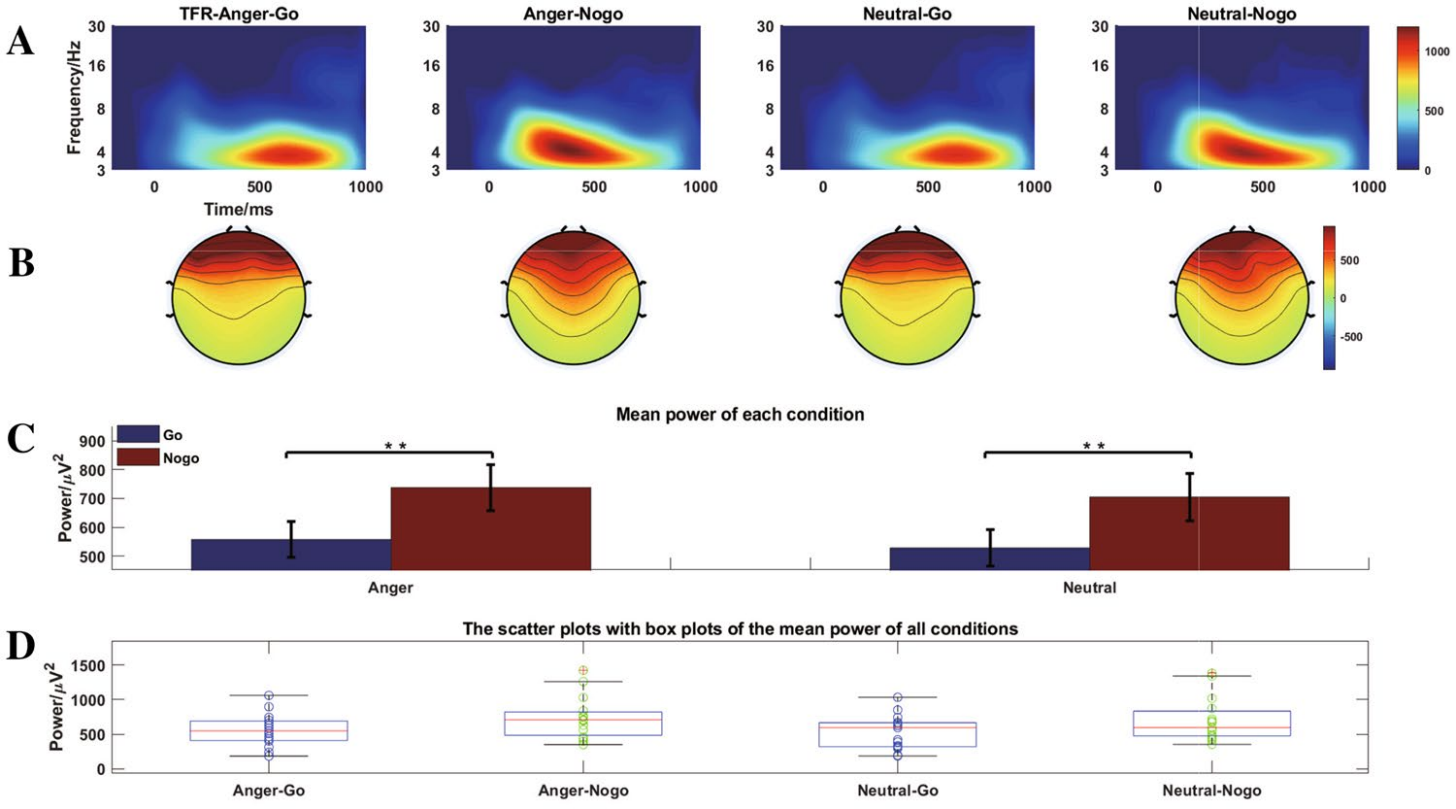
**a** The grand averaged time–frequency representations (TFRs). **b** Topographical distributions of the theta oscillations at Fz and FCz with the time window of 300–600 ms. **c** The mean power of every condition. **d** The scatter plots with boxplots of the mean power of every condition. Anger-Go, go task of the anger-associated words; Anger-Nogo, Nogo task of the anger-associated words; Neutral-Go, go task of the neutral words; Neutral-Nogo, Nogo task of the neutral words; '**' represents ${p < 0.01}$

Combining the previous studies (Benvenuti et al. 2017; Harper et al. 2014; Karakaş et al. 2000; Kirmizi-Alsan et al. 2006; Pandey et al. 2016) with the time–frequency representations (TFRs) and topographies of all conditions of the present data, we selected the Fz, FCz electrodes for analysis of the theta oscillation (range 3–7Hz) between 300 and 600ms. Then multivariate rm-ANOVAs were computed on theta oscillation using emotion (Anger and Neutral), task (Go and Nogo) as within-subject factors. Figure 4a–c displayed the grand averaged TFR of every condition at Fz and FCz, topography of the theta oscillation, and the corresponding mean power of every condition, respectively. In order to show the corresponding power of theta oscillation of every participant under each condition, the scatter plots with boxplots were displayed in Fig. 4d as well.

The results illustrated that the main effect of task was significant $( F_{(1,14)}= 10.378, p = 0.006, {\eta ^{2}_p} = 0.426)$. However, the interaction effect between emotion and task was insignificant $(\ F_{(1, 14)} = 0.007, p = 0.936, {\eta ^{2}_p} =0.001)$. Similarly, there was no significant main effect between anger and neutral condition $(F_{(1,14)} = 2.816, p =0.116,{\eta ^{2}_p} = 0.167)$.

Through visual inspection of the TFR and the mean power of each condition or stimulus for TFA results, the power of Nogo task ( mean:721.45 ${\upmu V}^2$; std: 78.81 ${\upmu V}^2$ ) was significantly higher than that of Go task (mean: 542.91 ${\upmu V}^2$; std: 62.40 ${\upmu V}^2$). In addition, the anger condition (mean: 647.70 ${\upmu V}^2$; std: 66.32 ${\upmu V}^2$) also elicited stronger power than that of neutral condition (mean: 616.65 ${\upmu V}^2$; std: 65.90 ${\upmu V}^2$).

In order to demonstrate that the statistical results are affected by the selected ROI for TFA compared with those of the proposed method, the ANOVA reults of another RIO (time window: from 200 to 700 ms; frequency range: 3–7 Hz) were also shown. The main effect of emotion $(F_{(1,14)} = 1.955, p = 0.1840, {\eta ^{2}_p} = 0.123)$ and interaction effect between the two factors $(F_{(1,14)} = 0.023, p =0.8810, {\eta ^{2}_p} = 0.002)$ were insignificant, respectively. In addition, there was a significant main effect for tasks $(F_{(1,14)} = 8.643, p = 0.0110, {\eta ^{2}_p} = 0.382)$. The methods, which can be used to precisely determine the ROI of TFR of every condition according to the corresponding boundary, were not discussed in this study.

### Multi-domain Features of NPLC

Aiming at extracting the NPLC of interest, the results from each step of the tensor decomposition analysis of ERP are as following.

Using the CFBW confirmed in “Selection of an Optimal Set of Parameters for CMCWT” section ($f_b = 1, f_c = 1$), the induced method was performed on all participants’ data for TFA. The sampling point is nonlinear distribution in the frequency domain, with 30 points set in the whole frequency band of interest (3–30 Hz).

The fourth-order tensor was formed by the time–frequency results.

**Fig. 5:**
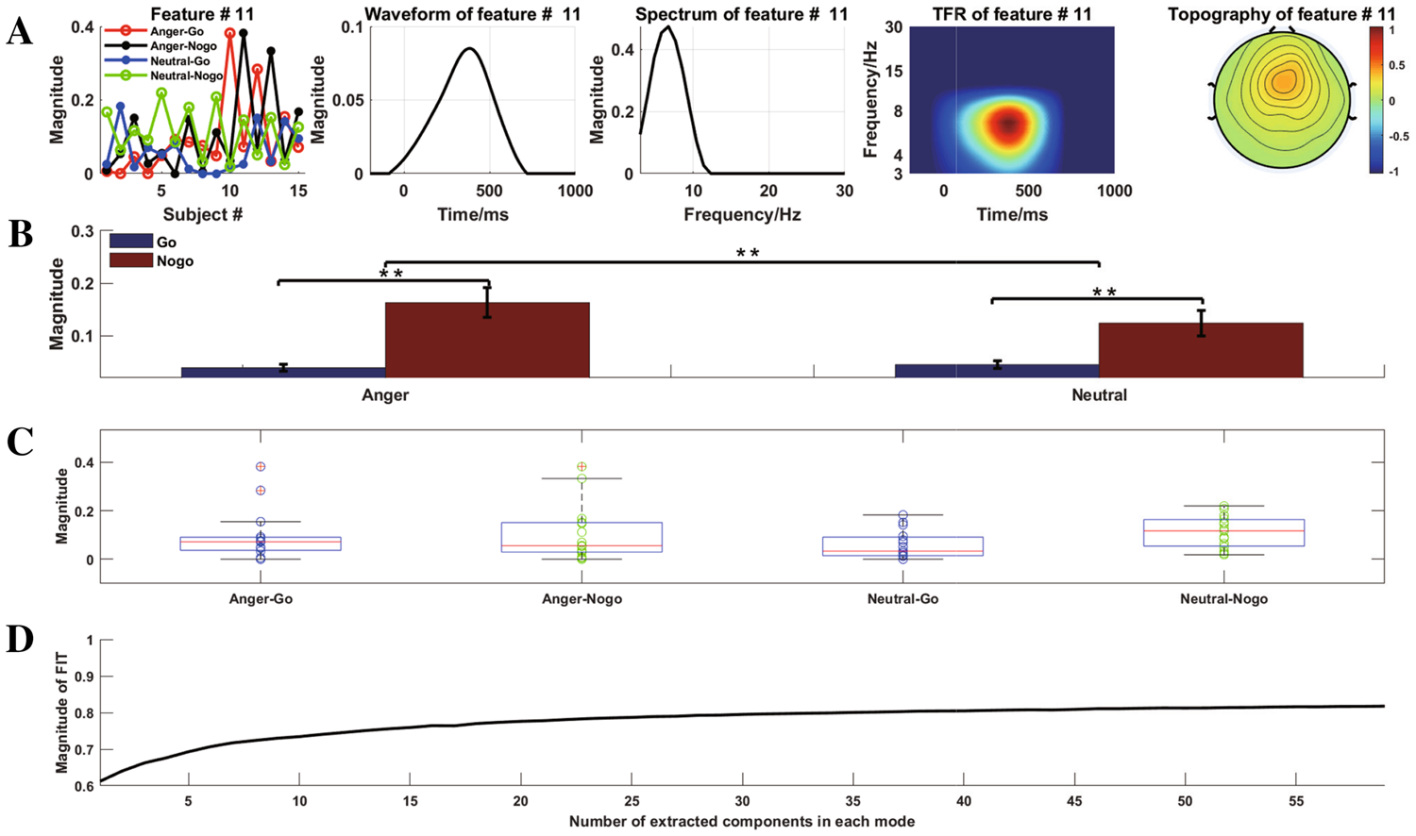
**a** Multi-domain features of NPLC of interest as well as the corresponding temporal, spectral, and spatial components were extracted from all brain activity. **b** The mean magnitude of every condition. **c** The scatter plots with boxplots of the mean magnitude of all conditions. **d** The magnitude of FIT, DIFFIT is performed on this curve. '**’ represents ${p < 0.01}$

According to the fit value as shown in Fig. 5d, 36 components were extracted in each mode. The detail of how to define the number of extracted components for CPD can be found in our previous studies (Cong et al. 2012c, 2013, 2014, 2012b).

We considered the temporal, spectral and spatial properties of NPLC of interest as shown in “Time–Frequency Analysis” section. Its latency fell in the range from 300 to 600 ms in the time domain, the peak of corresponding spectrum is below 8Hz, and its peak amplitudes are distributed in the frontal–central cortex in the space domain. The 11th component was chosen (in Fig.  5a). In addition, the TFR in Fig. 5a was based on the outer product of the temporal and spectral components.

When the multi-domain features were determined, two-way (emotion and task) repeated measurement statistical test were performed to investigate the between-task differences under emotion condition (Anger and Neutral) with 0.05 as the level of significance, and Greenhouse Geisser correction was performed when necessary. The results showed that the interaction effect between emotion and task reaches a significant level $( F_{(1,14)} =10.607,p = 0.006,{\eta } ^{2}_p = 0.431$). There was a significant main effect of both emotion $(F_{(1,14)} = 6.162, p = 0.026,{\eta } ^{2}_p = 0.306)$ and task $(F_{(1,14)} = 17.688, p = 0.001,{\eta } ^{2}_p = 0.558)$. Through post hoc analysis, the results demonstrated that the power of anger condition was larger than that of neutral stimuli in the Nogo task $(p =0.005)$, not in the Go task $(p = 0.367)$. By contrast, there was a significant main effect of task conditions under both anger $(p<0.001)$ and neutral condition $(p = 0.005)$. Thus, this study found that the power of NPLC oscillation obviously increases in anger words when compared to neutral words in the Nogo task as shown in Fig. 5b. In addition, the scatter plots with boxplots were also shown in Fig. 5c such that the feature of every participant in every condition can be observed.

## Conclusion and Discussion

Using CPD to separate the multi-domain features of NPLC of interest, this study investigated the differences between tensor decomposition analysis and TFA of ERP data. The tensor-based results were more discriminative than those derived from TFA. The method based on tensor decomposition showed not only the significant main effect of task condition, but also significant interaction effect between emotion and task. The main effect of emotion was found to reach a significant level. Moreover, the proposed method ensured that statistical analysis results donot change with ROI. This manifested that the derived features fulfill expectations in this study, and it should be fundamental for the extension of our proposed method for the analysis of other EEG/ERP data.

In this study, the time–frequency results are used by averaging the time–frequency transforms of each single-trial data to separate the multi-domain features of NPLC of interest, and it is different from those methods which obtain COI by averaging multi-trial waveforms in time domain and calculating the time–frequency transforms of averaged ERP data. Moreover, there are several methods to form a high-order tensor to extract the information of short ERPs data simultaneously in the time, frequency, space, and participants modes. For example, in order to study the properties of NPLC at the single-trial level, the fourth-order tensor can be comprised of frequency by time by channels by subjects-trials. Particularly, the third-order tensor is constructed including temporal, spectral, and spatial information, because the time-locked characteristics of NPLC in different single-trial are not deterministic. Few strictly time-locked characteristics of NPLC are preserved.

Furthermore, CPD is a group analysis method that performs on the high-order tensor of brain activity collected from different participants and stimuli/conditions (Zhou et al. 2016). It assumes that all subjects share the same information of components in the time domain, frequency domain, and space domain, while the variance in signatures of all participants is revealed by those common components (Cong et al. 2015a). As we all know, the EEG/ERP data of one subject in each condition/stimulus can form one block tensor. In other words, for one subject’s data, the block tensor can be a third-order tensor (time by channels by stimuli/conditions) or fourth-order tensor (frequency by time by channels by stimuli/conditions), so multi-participant data can form multi-block data. Therefore, coupled/constrained matrix and tensor factorizations can be applied to extract common and individual components and/or build links between them (Zhou et al. 2016).

There are several drawbacks for using CPD and TFA to extract the NPLC of interest from ERP data. One limitation is the small number of subjects were recruited to participate in the experiment in this study. The ERP data were only collected from 15 participants. Another one is the method to extract NPLC by tensor decomposition has not been employed in other ERP data. Additionally, in Fig. 4a, the grand averaged TFRs clearly display that the theta oscillation of interest of every condition has a specific and visible boundary and the ROIs of the four conditions are different. Hence, the same ROI for all conditions used for statistical analysis in the research is unreasonable and arbitrary. The techniques, such as edge detection method based on Canny, Marr–Hildreth, Deriche, Sobel, and Laplacian algorithms, can be used to precisely mark the edge of ERO of interest of every condition respectively in the TFR (Milanović et al. 2019). In this study, the tensor decomposition was used to extract the multi-domain features of NPLC simultaneously for an expected statistical results, evidencing that this method is promising with substantial potentials in neuroscience applications.
